# Michigan State Dental Association

**Published:** 1885-05

**Authors:** E. C. Moore


(TO BE CONTINUED.)

---

### MICHIGAN STATE DENTAL ASSOCIATION.

REPORTED FOR THE INDEPENDENT PRACTITIONER BY E. C. MOORE, D. D. S.

The regular annual meeting of this association was held at Detroit, on Thursday and Friday, March 26th and 27th, 1885.

The time was largely occupied by necessary routine business, but some of the discussions were very interesting and profitable.

Dr. Case read an interesting paper upon artificial vela, illustrated by drawings and demonstrated upon a living subject, a young man, who without the artificial velum was unable to articulate distinctly, and whose defect was especially apparent in his efforts to pronounce words containing the hard sounds of C, K, G and Q, and other sounds requiring a forcible expression, which defects were, to a great extent, overcome by the use of the artificial velum.

The subject of "Filling Teeth, Materials, Instruments, etc.," elicited a long discussion, of which the following is an abstract:

*Dr. Harroun*, of Toledo, Ohio—I line almost every cavity that I fill, unless it is a very small one, with oxy-phosphate of zinc, or something of that character. In proximal cavities I am using Dr. Robinson's fibrous filling, employing a burnisher, and not a serrated instrument for inserting it. If there is any masticating surface, I fill up as far as I wish with tin, and put gold on the outside. Thus far I have met with satisfactory success. The little sensitiveness that exists in many cases soon wears away, and in a week or so there is no trouble at all. This I attribute to the chemical action of the materials, while the tooth is in a better state of preservation than if it were filled only with gold. I am entirely opposed to the use of amalgam in any form for filling teeth. I have observed many cases of mercurial poisoning arising from amalgam fillings, where the general system was seriously affected. I have, in my own practice, had three cases of marked mercurial poisoning, all arising from amalgam fillings. The general system was affected, in one case producing a loss of voice by paralysis of the vocal organs. In another case, that of a lady, the teeth were sensitive, and her husband had filled them with amalgam, because of the difficulty in using any other material. She could not eat pickles, or take acids of any description, without disagreeable sensations. Her face, which before the fillings were put in was rosy and bright, was sickly and pale, and her system showed the traces of mercurial poisoning. I persuaded her to have the amalgam taken out of her teeth, and she was better in a short time; her face recovered its color, and she could eat pickles and acids. Another case was that of a man whose teeth were filled with amalgam. He was troubled with constant hacking and coughing, and his constitution seemed to be breaking down. I was satisfied from the peculiar taste he experienced when eating, that the fillings were the cause of the trouble. He consented to have them removed, and from the time of their removal he commenced to grow better, his food tasted better, and the bad symptoms disappeared. The general health is affected very often by amalgam fillings. There is a disposition, on the part of some members of the profession, to fill teeth for their patients according to the amount of money the patient is able to pay, without regard to whether it will injure them or not. No dentist should put amalgam, or any other bad material into a tooth, simply because he cannot get pay for anything better. The patient should be persuaded to have tin fillings. We should not put poison in the mouth because the patient cannot afford to pay for gold. The temptation may be strong; the practitioner may find it difficult to resist it; but for myself I cannot put in an amalgam filling and go to bed and sleep sweetly. It is a point of conscience. Still, if a dentist is convinced that he can put in an amalgam filling without doing harm, let him do it. I cannot, but I can put in a gold filling, a tin filling, or oxy-phosphate,or gutta-percha, and convince my patient that nothing will injure him, and at the same time feel satified with myself. The metallic materials which I use for filling teeth are gold and tin. I use tin-foil, or the fibrous tin of Dr. Robinson's make, or gold. Of course I use gutta-percha and the oxy-phosphates for temporary fillings. In all large crown cavities, where I have good anchorage, I line the cavity with oxy-phosphate or oxy-chloride of zinc, and put on a gold covering. As for the mode of introducing and condensing the fibrous filling, I use a strong, tapered burnisher. I place a sufficiency of the material into the bottom of the cavity, and press it against the uneven surface of the tooth; then I lay on another piece and burnish that down, and go on until I get it all in and it is an absolutely solid compound, burnished together. The adhesion is perfect. That filling will not turn black inside, any further than it is affected by oxidation from the outside. I have experimented and determined that the gases or secretions of the mouth may penetrate this fibrous material and change it to darker colors, but if it is burnished down that does not take place; it is all one solid mass.

*Dr. Taft*, of Ann Arbor —I think there is a disposition on the part of the profession to avoid the use of amalgams, or fillings in which mercury enters, and I am glad to observe this. I like the idea suggested by Dr. Harroun, of lining cavities, especially where there is a predisposition to sensitive dentine. There are various methods of doing this. Some simply use a lining made from a solution of gutta-percha, while others employ oxy-phosphates, or materials of that kind. The point is to secure to the living dentine that may be exposed in the walls of the cavity, as near the normal condition as possible. Avoid contact with foreign agents; avoid the influence of thermal change on the living dentine, which was not intended by nature to be exposed to these outside influences. This is the great objection to gold. Many failures occur by the transmission of thermal changes to sensitive dentine, so that its physiological action in respect to nutrition does not take place, and hence we rarely find under gold fillings, though they may have been there for years, any of the improvement or solidification of dentine which is found under many other fillings. This is one of the points that should be avoided in filling with gold. The tin will meet this objection to some extent, and I notice a greater disposition to experiment with a mixture of gold and tin than heretofore. From the experiments that I have made with tin and gold (I have not yet adopted this kind of filling as a mode of practice), I am free to confess that my expectations have been exceeded; they have gone beyond my former ideas in that matter, and my opinions have been somewhat modified. It is desirable to protect the walls of the cavities, and where there is sensitiveness to protect against thermal changes. The tin and gold mixture should be tried, and faithfully tried, and reported upon. I have experimented with Dr. Robinson's metal, and like it very much. Perhaps I do not know how to use it as I ought, but for me it is not so easily introduced as gold and tin-foil. I think this is my own fault. The mixture of this with oxy-phosphate I regard as a very good filling indeed. I change method and materials every now and then, seeking to find something better than that I have, but I have never blundered into the use of amalgam.

I think tin may be used with decided advantage upon the cervical borders of proximal cavities. These are very vulnerable to the recurrence of decay. The dentine at that point is very much exposed to decay-producing agents. It is one of the most difficult points to fill. In the preparation of the cavity there is very likely to be an irregularity of the cervical edge that cannot be completely filled. In the introduction of the filling the instrument may be driven down upon that, and if it had been left acute or sharp it may be fractured or broken, and there is always danger attached to that, even though it may be but a little comminution.

Another defect often found at this point is that the filling is too full, and not dressed down perfectly smooth with the tooth, so that when cleansing with a pick or a thread everything may not be removed from that surface. Then again it may drop back a little, leaving a little pocket, which affords good lodgement for foreign substances. With amalgam fillings this projecting condition is likely to occur. Sufficient care is not usually taken to dress them down. It may be that the amalgam expands, although it is asserted that it neither expands nor contracts. I think as many failures result from the neglect to properly finish fillings, as from anything else. However carefully it may be introduced, however perfectly the cavity may be sealed, if the surface is left rough upon them foreign substance will lodge and remain, to decompose and act upon the border of the dentine, and decay will often commence at this point, even though the fillings have been well introduced and the cavities thoroughly sealed.

As to the mode of introducing tin and gold together, experiments have been made in several directions. Some line the cavities with tin in the first place, in the manner that Dr. Blount, of Geneva, lines with gold. Some say that the lining should not come to the border of the cavity, while others assert that it should, if the tooth is to be protected. That is the method of Dr. Abbott, of Berlin. As to exposing tin in the mouth, the circumstances will determine whether this can be done. Where the secretions are not vitiated the tin will remain in good condition. The cavity may be lined with tin alone, or it may be mixed with gold. You may roll together a sheet of tin-foil and a sheet of soft gold foil, and fold them together and cut off strips, and use them in that way. I think that is the way Dr. Abbott uses them, and he also suggests that it may be rolled, and made into little cylinders and little blocks. As to the class of tin-foil, I have used numbers 6 to 10 more than any others. No. 4 is considerably thicker than No. 4 gold foil.

